# Artificial intelligence-enhanced biosurveillance for antimicrobial resistance in sub-Saharan Africa

**DOI:** 10.1093/inthealth/ihae081

**Published:** 2024-11-15

**Authors:** Innocent Ayesiga, Michael Oppong Yeboah, Lenz Nwachinemere Okoro, Eneh Nchiek Edet, Jonathan Mawutor Gmanyami, Ahgu Ovye, Lorna Atimango, Bulus Naya Gadzama, Emilly Kembabazi, Pius Atwau

**Affiliations:** Department of Research, Ubora Foundation Africa, Kampala 759125, Uganda; School of Public Health, University of Port Harcourt, River State 500001, Nigeria; Department of Community Medicine, David Umahi Federal University Teaching Hospital, Uburu, Ebonyi State 480101, Nigeria; Department of Community Health Ministry of Health; Akwa Ibom State, 520108, Nigeria; Global Health and Infectious Diseases Group, Kumasi Centre for Collaborative Research in Tropical Medicine, Kumasi GA107, Ghana; Department of Community Medicine, Jos University Teaching Hospital, Plateau State 930241, Nigeria; Department of Research, Ubora Foundation Africa, Kampala 759125, Uganda; Department of Community Medicine, College of Medical Sciences, Abubakar Tafawa Balewa University, Bauchi State, 740272, Nigeria; Department of Pharmacology and Therapeutics, School of Biomedical Sciences, Makerere University, Kampala 10207, Uganda; Center for Biomedical Engineering, India Institute of Technology, New Delhi 600036, India

**Keywords:** AMR, AMR biosurveillance in SSA, AMR-biosurveillance, AMR-technology, artificial intelligence, biosurveillance

## Abstract

Antimicrobial resistance (AMR) remains a critical global health threat, with significant impacts on individuals and healthcare systems, particularly in low-income countries. By 2019, AMR was responsible for >4.9 million fatalities globally, and projections suggest this could rise to 10 million annually by 2050 without effective interventions. Sub-Saharan Africa (SSA) faces considerable challenges in managing AMR due to insufficient surveillance systems, resulting in fragmented data. Technological advancements, notably artificial intelligence (AI), offer promising avenues to enhance AMR biosurveillance. AI can improve the detection, tracking and prediction of resistant strains through advanced machine learning and deep learning algorithms, which analyze large datasets to identify resistance patterns and develop predictive models. AI's role in genomic analysis can pinpoint genetic markers and AMR determinants, aiding in precise treatment strategies. Despite the potential, SSA's implementation of AI in AMR surveillance is hindered by data scarcity, infrastructural limitations and ethical concerns. This review explores what is known about the integration and applicability of AI-enhanced biosurveillance methodologies in SSA, emphasizing the need for comprehensive data collection, interdisciplinary collaboration and the establishment of ethical frameworks. By leveraging AI, SSA can significantly enhance its AMR surveillance capabilities, ultimately improving public health outcomes.

## Introduction

**Figure 1.**
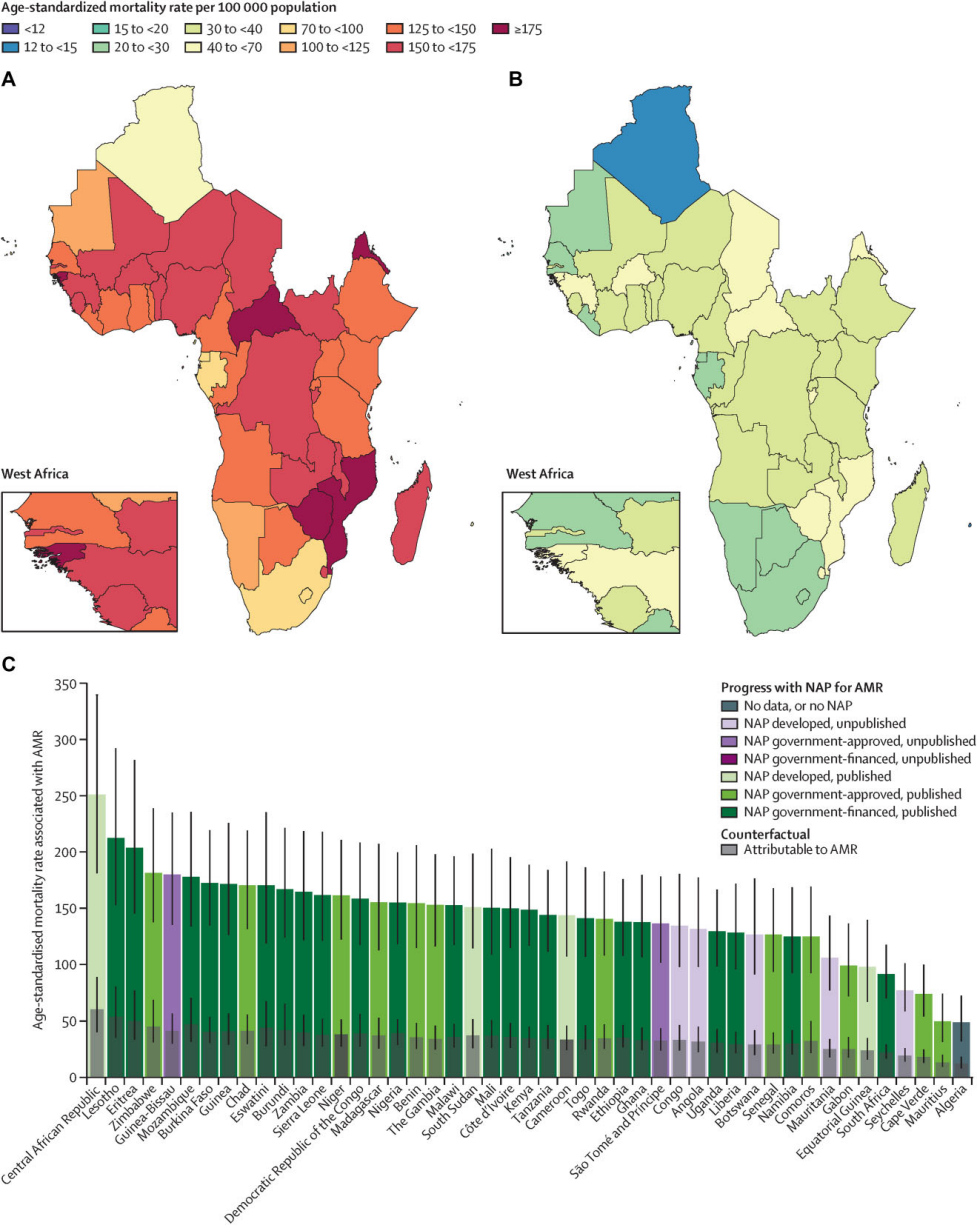
Country-based age-standardized mortality rates associated with AMR per 100 000 population.^3^ AMR: antimicrobial resistance; NAP: national action plan.

Globally, antimicrobial resistance (AMR) continues to ravage different individuals and healthcare systems. AMR is a condition that arises following the failure of antimicrobials to effectively combat different microbial agents, such as bacteria, parasites and viruses. By 2019, >4.9 million fatalities and 1.27 million mortalities were attributed to AMR worldwide.^1^ Sadly, forecast projections estimate that AMR may contribute to about 10 million mortalities annually by 2050 in the absence of appropriate interventions.^2^ AMR has significantly affected low-income countries, with an estimated 3.83 million deaths arising from infections, of which 1.05 million deaths were AMR associated by 2019. Furthermore, approximately 250 000 deaths of the 3.83 million were attributed to AMR, indicating a serious public health concern.^3^ Africa alone had the highest mortality attributed to AMR, of approximately 27.3 deaths per 100 000 population.^4^ Notably, countries such as the Central African Republic, Lesotho and Eritrea had >200 age-standardized mortalities associated with AMR per 100 000 population. Compared with this, Mauritius and Algeria had <50 cases per 100 000 population.^3^ Other African countries reported a variable number of cases, as demonstrated in Figure 1.

**Figure 2.**
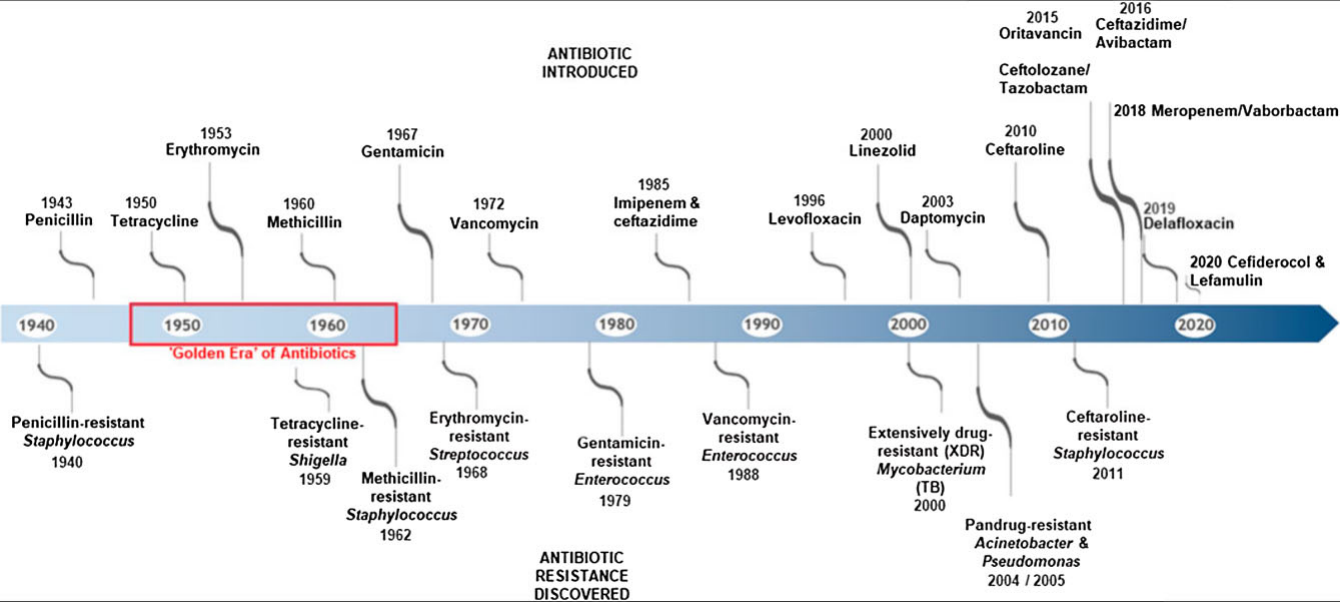
Antibiotic discovery and the development of antibiotic resistance.^2^

Different microbes have dominated resistance patterns, such as the Staphylococcus strains whose resistance to penicillin started as early as 1940. Other resistant microbes, commonly known as ‘superbugs’, include *Streptococcus, Shigella, Enterococcus, Mycobacterium, Escherichia coli, Acinetobacter* and *Pseudomonas*. These microbes have been resistant to conventional antimicrobials such as methicilin, vancomycin, ceftaroline and gentamicin.^2^ Evidence demonstrates that the resistance patterns noted among antimicrobials occur shortly after discovering a specific antimicrobial agent. For example, ceftaroline, a novel fifth cephalosporin class discovered in the early 2010s, was no longer effective for staphylococcus strains by 2011.^2^ Additionally, microbial agents such as levofloxacin (discovered in 1996) and linezolid (discovered in 2000), were no longer effective with some mycobacterium strains, resulting in the development of extensively drug-resistant mycobacterium TB, as demonstrated in Figure 2.^2^ In perspective, >3.5% of active global TB cases globally were classified as multidrug-resistant cases by 2023.^5^ These statistics significantly demonstrate a global burden that necessitates immediate intervention from multiple perspectives. Even although high-quality AMR surveillance is the hallmark of easily identifying and combating AMR, sub-Saharan Africa (SSA) still grapples with obtaining sufficient information necessary for the fight.^6^

Yet, technological advancement has offered various opportunities to leverage healthcare improvement. With the development of artificial intelligence (AI), a facet of technological advancement, multiple avenues of improving healthcare outcomes have been explored. These include disease modeling, telemedicine, enhanced digital communication, continuous health monitoring using monitoring devices and precision medicine.^7–9^ AMR biosurveillance entails the collection, analysis and communication of different AMR information collated into relevant data to improve measures combating AMR. Further, it involves a detailed analysis of microbial populations to easily identify resistant strains and develop immediate action plans.^10^ In 2015, the WHO unveiled the Global AMR Surveillance System (GLASS), focusing on streamlining collected information about AMR.^6^ Various surveillance systems sprung from the GLASS project, such as “Central Asia and Eastern Europe” (CAESAR), “Red Latino Americana de Vigilancia de la Resistencia a Los Antimicrobianos” (ReLAVRA), and “European Antimicrobial Resistance Surveillance Network” (EARS-Net) which have sufficiently provided AMR information in these regions they operate in..^6,11,12^ SSA still lacks a robust surveillance network to provide sufficient information, leading to the availability of fragmented and incomplete data.^6^ However, some African countries have developed national surveillance frameworks to continue the fight against AMR; for example, in 2019, Burkina Faso implemented a surveillance system based on international standard operating procedures in 15 laboratories.^13^ AI development offers the potential of overcoming AMR surveillance barriers, enhancing the fight against AMR. This review explores the available literature concerning AI-enhanced biosurveillance methodologies from the perspective of SSA and how AI utilization can enhance the fight against AMR.

## Methodology

We searched scholarly articles from different databases such as PubMed, Scopus, Google Scholar, Medline and Oxford University Press. Different search words, phrases and strings such as ‘AMR’, ‘global burden of AMR’, ‘AMR surveillance’, ‘AMR AND AI’, ‘AI and the future of AMR surveillance’ and ‘AMR surveillance in SSA’, among others, were used. Studies published after 2014 were included in this review to obtain the latest information. The studies obtained were carefully reviewed to ensure that they had the information relevant to the review. To augment our search strategy, gray literature searches were made in different libraries such as The WHO directory, The Global Health Network, the Centers for Disease Control and Prevention, the World Bank and the Fleming Fund Directory.

## The role of AI in biosurveillance

AI is a technological component involving the development and improvement of computer systems to perform various tasks with higher precision analogous to human intelligence. Some of these tasks can include problem-solving, decision-making and learning. In the context of biosurveillance, AI can improve the detection, tracking and prediction of various resistant pathogenic strains, prompting the development of effective remedies.^14^ AI algorithms, such as machine learning (ML), deep learning and natural language processing can be employed in pattern recognition by analyzing large datasets about microbes and microbial management from different African countries.^15^ Analysis of these datasets can inform stakeholders about the patterns and anomalies in AMR trends, allowing for the early detection of more resistant strains. Further, using the information obtained from these datasets, AI can build different predictive models that can forecast resistant pathogens.^16^ These forecasts enable the development of multiple proactive measures to prevent further outbreaks of resistant strains and optimize proper resource allocation. Furthermore, AI can be applied in genomic analysis to identify potential genetic markers and AMR determinants among specific populations.^17^

Besides, AI can significantly improve decision-making obtained from the optimal treatment strategies developed by the different algorithms. Biomarker methods such as multiple logistic regression also use AI techniques; by considering multiple biomarkers and continuous likelihood ratios, AI-based approaches have also been used in accelerating the discovery and development of antimicrobial peptides (AMPs) by utilizing quantitative structure-activity relationship descriptors and other physical and chemical properties to identify novel peptides with antimicrobial activity.^18^ AI technologies have also been used to predict the synergy of drug combinations via methods such as drug combination response prediction. AMPs and antibiotic combinations offer potential alternatives to conventional antibiotics, thereby offering a means of combating antibiotic resistance.

**Figure 3.**
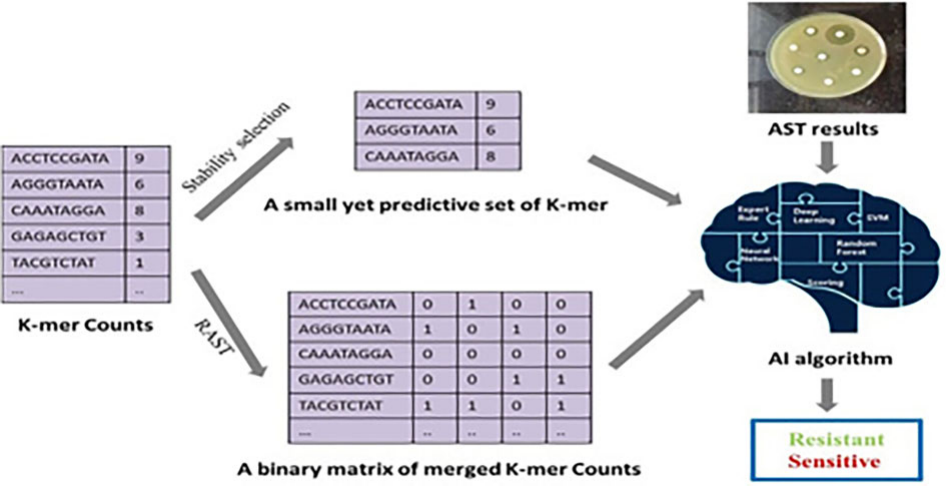
A simple model integrating AI in the WGS-AST methodology for predicting AMR.^20^ AI: artificial intelligence; AMR: antimicrobial resistance; AST: antimicrobial susceptibility testing; WGS-AST: wide genome sequencing for AST.

Additionally, AI in AMR-biosurveillance can be used in various methods, including AMR prediction, detecting the rational use of antibiotics, exploring AMPs and evaluating antibiotic combinations.^15^ AI has significantly advanced traditional antimicrobial susceptibility testing (AST) methods, which are critical for determining the resistance of bacteria to antibiotics. For instance, a combination of AI and a flow cytometer-AST technique coupled with supervised ML, adopted by Inglis et al., achieved rapid results and reduced the testing time to minutes, thereby streamlining the AST process.^19^ Furthermore, AI has also been pivotal in refining wide genome sequencing for AST (WGS-AST) through leveraging whole-genome data to predict AMR, as demonstrated in Figure 3. Despite these advantages, there is a deficiency of primary data about the utilization of AI algorithms in combating AMR from the African perspective, warranting an intervention.

## AI algorithms for AMR prediction and detection

**Figure 4.**
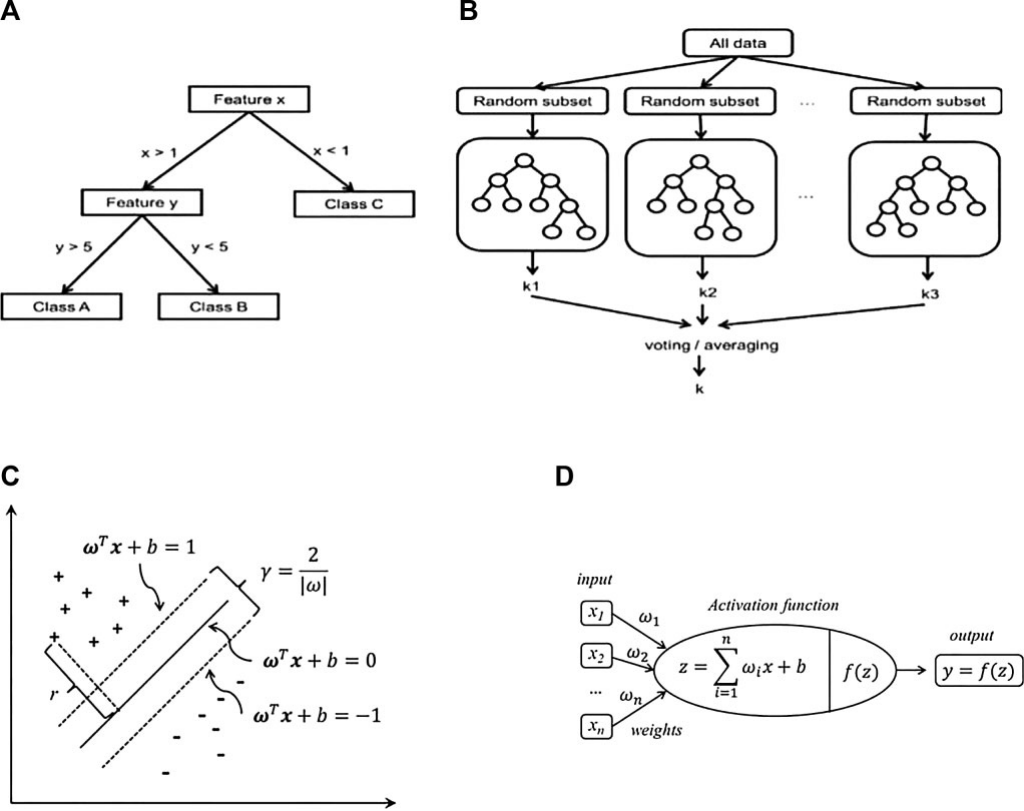
The different AI algorithms. (A) A simple decision tree with three cluster levels; (B) a random forests demonstration schema; (C) a simple vector machine with two clusters of the dataset; (D) representation of an artificial neural network.^20^ AI: artificial intelligence.

As AI is integrated into AMR prediction and detection, various algorithms have been developed that can be applied differently. However, these have been extensively utilized in high-income countries, while SSA nations have not explored these algorithms in AMR surveillance. Naïve Bayes (NB), decision trees (DT), random forests (RF), support vector machines (SVMs) and artificial neural networks (ANNs) are some of the most commonly used AI algorithms.^20–22^ These algorithms are ranked based on their interpretability and speed, with NB being the best and easiest to interpret; however, each algorithm has its advantages and disadvantages, requiring different applicability situations. An illustration of these algorithms and their respective equations is demonstrated in Figure 4. NB uses the Bayes’ theorem with independent assumptions of each other; RF utilizes two key concepts (i.e. random sampling from the training set and a random subset of features to improve accuracy and reliability); and DT is a classification algorithm used for decision-making in allocating medical resources adequately through assessing the AMR burden.^20^ ANNs use modeled neurons in the human brain to transmit information, while the SVM is a binary classification model used to separate samples into different classes to predict AMR phenotypes, whether resistant or susceptible.

Furthermore, ML and predictive analytics are revolutionizing the field of AMR by enabling the discovery of novel antimicrobials and elucidating the mechanisms through which resistance occurs. ML utilizes various models including RF, SVM, and ANNs, to analyze genomic data including WGS, pan-genomes and core genomes to predict AMR phenotypes or continuous AST values. Interpretable ML models are also being developed to go beyond the ‘black-box’ nature of conventional algorithms, allowing direct insights into biological mechanisms.^23–25^ This is crucial for AMR, where understanding the molecular basis of resistance can lead to targeted interventions. For instance, ML models that integrate genomic data from clinical isolates with metabolic network models have identified novel resistance mechanisms specific to certain antibiotics. These precise models offer a potential solution for AMR biosurveillance in SSA, a region that is just adapting to AI utilization in healthcare.

## Current biosurveillance methods for AMR

Currently, two major methods are used for diagnosing AMR: AST and molecular methods, such as WGS-AST. AMR surveillance depends on the qualitative measure needed to compare different microbial populations. This could be defined based on phenotypic thresholds, inhibitory zones and gene-centric peculiarities.^26^ AST is a culture-based method that aids in determining which antibiotics are effective against a microbe of concern and what level of doses during therapy should be administered. Examples of traditional AST procedures include disk diffusion, broth microdilution, gradient tests, breakpoint tests and agar dilution. These procedures depend on exposing bacterial isolates to a series of antimicrobials and visually detecting their growth.^27^ Advanced culture-based techniques involve the use of automated AST systems, which have diverse antimicrobial panels tailored for pathogens that are both Gram-negative and Gram-positive.^28^ They use modern optoelectronic systems, fiber optics, microfluidics and indicator dyes that are sensitive to the redox state or pH to improve the sensitivity and overall performance of optical systems used for testing purposes.^29^ Examples are Alfed 60 ASTTM, Microscan WalkAway, BD Phoenix Automated Microbiology System, Vitek 2 System and Sensititre ARIS 2X.^27^

Molecular-based techniques can accurately identify and detect antibiotic resistance genes, an advantage they have over phenotypic assays. These techniques include nucleic acid amplification testing (PCR, multiplex PCR, reverse transcriptase PCR, real-time PCR and isothermal amplification methods), genomic sequencing and metagenomics, DNA microarray and fluorescence in situ hybridization. Molecular-based techniques have limitations in their inability to determine minimum inhibitory concentrations and the possibility of missing some antibiotic resistance genes due to their limited sensitivity and coverage.^27^ The mass spectrometry-based methods (Matrix-assisted laser desorption/ionization–time-of-flight mass spectrometry-based and intact cell mass spectrometry (ICMS), biosensor-based AST system measures 16S rRNA molecules to evaluate bacterial growth in Hybridization-Based Systems.^27^ It combines nanotechnology, a plastic micro-electromechanical system and microfluidics with species-specific probes. Notably, the bioinformatics approach for detecting AMR genes and databases is used to make predictions or reveal formerly unidentified biological phenomena through different computer methodologies, including sequence and structural alignment and analysis of extensive biological datasets (genetic sequences, cell populations and protein samples).^30^ The GLASS was launched in eight low- and middle-income countries (LMICs; Bangladesh, Cambodia, India, Laos, Nepal, Pakistan, Thailand and Vietnam) by the WHO to facilitate a standardized approach for AMR surveillance worldwide, providing surveillance and laboratory guidance, tools and support to national AMR surveillance systems, with the objectives of standardizing approaches for data collection, data analysis and sharing of data globally.^31^ However, SSA still has significant strides to take in terms of utilizing these standardized tools and availing real-time information about AMR.

## Data sources and collection methods

AI-enhanced biosurveillance systems leverage diverse data sources, including clinical, microbiological, genomic and environmental data, to provide comprehensive coverage and insights into AMR patterns.^32^ The use and integration of these data sources in AI-enhanced biosurveillance for AMR shows their importance in enhancing surveillance capabilities and informing antimicrobial stewardship efforts. Clinical data serve as the foundation of AI-enhanced biosurveillance, providing valuable insights into patient demographics, antimicrobial use patterns and disease outcomes.^33^ Electronic health records, laboratory reports and prescription data are commonly used sources of clinical information.^34^ Studies have demonstrated that integrating clinical data into biosurveillance systems enables the identification of trends in AMR, facilitates early detection of outbreaks and supports evidence-based decision-making in clinical practice.^35^ Besides, microbiological data, including laboratory test results and antimicrobial susceptibility profiles, provide crucial information about the prevalence and distribution of resistant pathogens. By analyzing microbiological data, researchers can identify emerging resistance patterns, track the spread of resistant strains and assess the effectiveness of antimicrobial treatments. Evidence further demonstrated that integrating microbiological data into biosurveillance systems significantly improves the accuracy and timeliness of AMR detection, enabling more targeted interventions and resource allocation.^36,37^

From a clinical assessment, resistance trends automatically calculated by ARTEMIS had a strong positive correlation with EARS-Net and the Sentinel Surveillance of Antibiotic Resistance in Switzerland (SEARCH) systems. Furthermore, mean resistance rates extracted by ARTEMIS were not significantly different from those of either EARS-Net or SEARCH.^38^ Genomic data analysis plays a key role in understanding the genetic mechanisms underlying AMR. WGS technologies enable comprehensive analysis of bacterial genomes, enabling the identification of resistance genes, mutations and mobile genetic elements. Research has demonstrated that the integration of genomic data increases the ability to trace the transmission routes of resistant strains, predict antimicrobial susceptibility and guide the development of new therapeutic strategies.^36^ Finally, environmental data provide information on the broader ecological factors that influence AMR, including antibiotic use in agriculture, wastewater surveillance and antibiotic residues in food products.^39^ By integrating environmental data into biosurveillance systems, researchers can identify environmental hotspots of resistance transmission and assess the impact of environmental factors on the development of resistance. This emphasizes the importance of incorporating environmental data into surveillance frameworks to improve understanding of environmental drivers of AMR and inform specific interventions.

## Challenges, limitations and future directions of AI-enhanced biosurveillance

Implementing AI in biosurveillance can be hindered by different challenges, especially in the African context. SSA often lacks the large and high-quality datasets required by the different AI algorithms to produce meaningful predictions, assumptions and conclusions about AMR.^40^ Currently, most of the datasets about AI AMR available are from high-income countries (Table 1), leaving SSA at a deficit in utilizing AI with AMR information. Relying on AI trained on data from high-income countries in African settings risks introducing unintended bias against the African populations and may not offer accurate conclusions. Besides, AI models used for AMR surveillance can inherit biases from the data they are trained on.^41^ If these data primarily come from high-income countries, they might not accurately reflect AMR trends in LMICs. These biases can set in during data collection, labeling and model design, impacting the effectiveness of AI across different populations. The absence of large datasets creates an opportunity to enhance data collection methodologies in SSA to ensure data availability. The data can then be used to build tailored large language models that can be effectively utilized against AMR.

**Table 1.** The different AI-AMR available datasets^21^

Name	Description	Website
PATRIC	Bacterial genomes with AMR phenotypes and minimum inhibition concentration	https://patricbrc.org/
CARD	ARG and their resistance mechanisms	https://card.mcmaster.ca/
ARDR	ARG information	https://ardb.cbcb.umd.edu/
BacMet	Antibacterial biocide and metal resistance genes	http://bacmet.biomedicine.gu.se
ARG-ANNOT	ARG and point mutations	http://www.mediterranee-infection.com/article.php?laref=282&titer=arg-annot

AI: artificial intelligence; AMR: antimicrobial resistance; ARG: antibiotic resistance gene.

Ethical issues, including obtaining traditional informed consent, become difficult when using complex AI models in healthcare and biosurveillance, particularly black-box algorithms. Clinicians may struggle to explain these models to patients. This raises the question of how much transparency is necessary for ethical patient interaction.^42^ The WHO provides a framework for ethical AI development in healthcare. However, there is still a significant gap concerning the implementation and utilization of these frameworks in different African countries; nevertheless, the USA and Canada, as well as the European Union, have already established regulations based on this framework. Furthermore, public records from the African context may not indicate existing regulations for AI use in healthcare within these regions. This highlights the need for African countries to consider developing appropriate AI regulations to ensure the ethical and responsible implementation of these technologies in their healthcare systems.^43^ However, some of these strategies, like the development of regulations, may require various resources, such as funds, which may also be limited based on the different healthcare budgets and healthcare financing available. Thus, African countries must devise economically friendly strategies. AMR biosurveillance is also limited by weak laboratory infrastructure, limited and poor staff capacity and training, poor communication between the medical team and laboratory personnel, low-quality assurance, as well as inadequate funding and lack of self-sufficiency in some African countries.^44^ Interdisciplinary collaboration between experts in medicine, microbiology, epidemiology, data science and AI is essential for addressing AMR from the African perspective. Bringing together diverse perspectives can lead to innovative solutions and comprehensive surveillance strategies. However, there are still insufficient data concerning the efficacy of these collaborations from the African perspective, creating another avenue for future research. Additionally, data sharing and integration of diverse data sources, including genomic data, clinical records, environmental data and surveillance data, can provide a more holistic understanding of AMR dynamics. Creating platforms, such as African regional databases for secure data sharing and interoperability, is crucial for facilitating collaboration and enabling comprehensive surveillance efforts.^45^

## Conclusions

AMR is still a huge global health challenge, with devastating impacts predicted if effective measures are not implemented. The burden is still severe in low-income regions, notably SSA, where inadequate surveillance systems are still significant, and these often lead to fragmented and incomplete data. AI offers transformative potential in enhancing AMR biosurveillance through multiple algorithms that can detect, track and predict resistant strains, such as RF, ANNs and SVMs. However, the successful integration of AI in SSA is still limited due to multiple challenges. For example, the region often lacks the large, high-quality datasets required for building robust AI models and mostly relies on data from high-income countries. Addressing this necessitates improved data collection and the development of tailored AI models suited to SSA's unique context. Ethical considerations also pose significant challenges. The complexity of AI models can make obtaining informed consent difficult. While global frameworks for ethical AI in healthcare exist, their implementation in SSA remains limited. Thus, developing and adhering to these ethical guidelines is crucial to ensure responsible AI deployment. Data sharing and integration of diverse data sources are also critical to provide a holistic understanding of AMR dynamics from the African perspective. Creating secure, interoperable platforms for data sharing within the region can facilitate collaboration and improve surveillance efforts. Notably, AI has the potential to revolutionize AMR biosurveillance in SSA, but realizing this potential requires addressing significant challenges related to data quality, ethical implementation and infrastructural limitations.

## Data Availability

Information in this review has been obtained from existing published studies thus data availability is not applicable in this scenario.
